# Deciphering the antiviral nature of endophytic *Bacillus* spp. against groundnut bud necrosis virus in cowpea and tomato

**DOI:** 10.3389/fmicb.2024.1410677

**Published:** 2024-06-06

**Authors:** M. Gayathri, R. Sharanya, P. Renukadevi, S. Nakkeeran, N. Saranya, S. Varanavasiappan, M. Raveendran, A. Suhail, Saad Alkahtani

**Affiliations:** ^1^Department of Plant Pathology, Centre for Plant Protection Studies, Tamil Nadu Agricultural University, Coimbatore, India; ^2^Agriculture College and Research Institute, Kudumiyanmalai, Pudukottai, India; ^3^Department of Plant Molecular Biology & Bioinformatics, Centre for Plant Molecular Biology and Biotechnology, Tamil Nadu Agricultural University, Coimbatore, India; ^4^Department of Plant Biotechnology, Centre for Plant Molecular Biology and Biotechnology, Tamil Nadu Agricultural University, Coimbatore, India; ^5^Department of Zoology, College of Science, King Saud University, Riyadh, Saudi Arabia

**Keywords:** GBNV, bacterial endophytes, virus titer, ELISA, real-time PCR

## Abstract

Tomato, the important vegetable crop, is severely affected by *Orthotospovirus arachinecrosis* which impacts heavy economic losses. The application of insecticide to manage viral diseases is not an environmentally safe approach. In view of these issues, we investigated the antiviral efficacy of 21 bacterial endophytes against GBNV in local lesion host (Cowpea-VBN3). Based on the reduction in lesion number and virus titer as estimated through both DAC ELISA and qPCR in cowpea, the bacterial endophytes viz., *Bacillus licheniformis* Soya1, *Bacillus tequilensis* NBL6, and *Bacillus velezensis* VB7 were selected and further tested in tomato. The study revealed the well-defined antiviral efficacy of these endophytes against GBNV. The percentage of disease incidence ranged from 16 to 24% in endophyte-treated tomato plants compared with untreated plants (88%). In addition, symptom severity was reduced, and the application of endophytes also in promotion of the growth compared with untreated control. DAC ELISA revealed that the tomato plants treated with bacterial endophytes challenged with GBNV showed reduction in the virus titer (0.26–0.39 @ OD 405 nm) at different days of interval after inoculation (0, 5, and 10 days) compared with untreated control (3.475 @ OD 405 nm). Additionally, reduction in the viral copy number in bacterial endophyte-treated plants was evident by real-time PCR. Furthermore, tomato plants bacterized with endophytes depicted significant correlation and reduction in viral load and disease incidence as revealed by the principal-component biplot analysis. Thus, the application of bacterial endophytes has a potential role in reducing the disease incidence, severity, and titer value of GBNV, which will be the promising management approach in future to mitigate the virus infection in tomato plants.

## 1 Introduction

Tomato is one of the most highly consumed vegetables in the world and provides significant economic and nutritional benefits to both growers and consumers. It is popular and versatile and contains various health-promoting compounds (Ali et al., 2020). Due to its status as a basic ingredient in a large variety of foods, tomato is grown worldwide for local use and export. Worldwide production of tomato was 186.11 million tonnes (mT) in 2021, while China is a global leader in its production followed by India with an average annual production of 20.69 mT (11.12% of world production) (FAOSTAT, 2024). The matter of concern in India is its productivity which is considerably low because of its susceptibility to major diseases. Among the diseases, bud necrosis disease (BND) caused by groundnut bud necrosis virus (GBNV) poses the greatest risk to tomato production, which causes yield losses upto 100% (Kunkalikar et al., 2011) depending on the stage of infection. In India, the bud necrosis disease was first reported in 1964 from Nilgris (Todd et al., 1975), and the causal agent was characterized as tomato spotted wilt virus (Ghanekar et al., 1979). However, in 1992, based on serology and host range studies, it was resolved that bud necrosis disease was caused by a tospovirus different from TSWV, which was characterized as groundnut bud necrosis virus (GBNV) (Mandal et al., 2012). Three tospoviruses including groundnut bud necrosis virus (GBNV), groundnut yellow spot virus (GYSV), and watermelon bud necrosis virus (WBNV) infect various vegetables in India, including tomato, potato, chilli, peppers, and watermelon (Mandal et al., 2012). GBNV, a member of the genus *Orthotospovirus* is quasi spherical in shape with a diameter of 80–120 nm and enveloped with tripartite genome of L, M, and S RNA. L RNA (8.9 kb) is negative sense, encoding the replicase protein (Rep), while M RNA (4.8 kb) is ambisense, encoding two proteins, glycoprotein (G1, G2) and movement protein (Nsm). Additionally, S RNA (3.05 kb) is also ambisense, encoding nucleocapsid protein (N) and non-structural small protein (NSs) (Satyanarayana et al., 1998). In recent past, *Orthotospovirus* infections are common, particularly in the Indian states viz., Tamil Nadu, Karnataka, Maharashtra, and Andhra Pradesh (Rai et al., 2020). According to Umamaheswaran et al. (2003), the crop is most vulnerable during flowering and fruit production stages. Early infection resulted in chlorotic and necrotic lesions on the leaves and drying of young buds followed by stem necrosis and severe stunting. Necrotic ringspots in unriped fruits and chlorotic ringspots in ripened fruits were typically observed at the later stages of infection (Raja and Jain, 2006; Basavaraj et al., 2017).

Management of viruses is a challenging task across the globe as only very few options are currently available for managing viral diseases in crop plants. Application of insecticide and other chemicals is inevitable, but indiscriminate usage has led to the development of resistance in insects apart from the potential risk of virus recombination. However, time is needed to identify the potential bioagents with beneficial activity and environmental safety. Combination of these antagonistic bacterial strains that are effective against viral infection is expected to activate immune responses. In addition, antiviral metabolites may also contribute to immunity against viral diseases. Beneficial bacteria, significantly aid in the mobilization of nutrients, trigger plant growth and activate defense mechanism against various diseases (Meena et al., 2017). Li et al. (2016) described the application of *Enterobacter asburiae*-inducing resistance against tomato yellow leaf curl virus in tomato. Under artificial conditions, various bacterial strains, including *Pseudomonas aeruginosa*, *Burkholderia* sp., and *Bacillus* sp., were well explored against virus diseases (Zehnder et al., 2000; Ramzan et al., 2016). Similarly, the application of *Bacillus* spp. has also triggered immunity and activated defense against tobacco streak virus in cotton (Vinodkumar et al., 2018) and PVY in potato (Amin et al., 2023). These studies paved a way for the current investigation to identify the potential bacterial endophytes and assess the efficacy against GBNV infecting tomato.

## 2 Materials and methods

### 2.1 Virus source

Tomato samples infected with groundnut bud necrosis virus exhibiting necrotic ring spots on leaves and stem necrosis were collected from major tomato-growing areas of Devarayapuram, Bolumvampatti, Marchinaickenpalayam, and Tholampalayam of Coimbatore district and Hosur region of Krishnagiri district, Tamil Nadu, India. The GPS data of each location are presented in Supplementary Table S1.

### 2.2 Detection of GBNV in field-collected samples through RT-PCR

Five samples (one representative sample per location) were subjected to RT-PCR analysis. Total RNA was extracted using the Trizol-phenol-chloroform method (Sigma-Aldrich) (Chomczynski and Sacchi, 1987). cDNA was synthesized from RNA using SuPrimeScript RT Premix cat no SR-2000 (GENETBIO Inc., Korea). The reaction mixture consisted of 10 μL of SuPrimeScript RT Premix, 1 μL of random primer, 4 μL of nuclease-free water, and 5 μL of total RNA. Contents were mixed gently, and the above reaction mixture was incubated at 50°C for 60 min followed by 70°C for 10 min. PCR was performed using the specific primer pairs for N gene, GBNV N-F 5′ATGTCTAACGT(C/T)AAGCA (A/G)CTC 3′ and GBNV-N-R 5′TTACAATTCCAGCGAAGGA CC 3′, to amplify the complete nucleocapsid gene (N) (size ~830 bp) of GBNV (Satyanarayana et al., 1998). Healthy tomato leaf was served as negative control. Amplification was performed in a thermocycler (Eppendorf Mastercycler nexus gradient S-Eppendorf, AG Hamburg, Germany) and programmed for one cycle of 5 min as initial denaturation at 94°C and 35 cycles involving 30 s of denaturation at 94°C, 1 min of annealing at 52°C, 2 min for extension at 72°C followed by one cycle of final extension for 10 min at 72°C. The PCR products were visualized at 1% agarose gel electrophoresis, and PCR products were sequenced at M/S Syngenome Lab Pvt. Ltd., Coimbatore. The sequence data were assembled using Bioedit 2.0 and analyzed through a BLAST search in the NCBI database.^1^ The N gene of the study isolates was compared with GBNV isolates retrieved from the NCBI database.

### 2.3 Maintenance of virus inoculum

Tomato plants with characteristics of GBNV symptoms were collected from the field which served as the source of inoculum. The virus inoculum was maintained in different indicator hosts, viz., *Vigna unguiculata* (VBN 3), *Gomphrena globosa*, *Chenopodium amaranticolor*, *Chenopodium quinoa*, and *Nicotiana benthamiana* through sap inoculation. Mechanical inoculation of GBNV was performed by maceration of approximately 1 g of infected tomato samples using 0.01 M sodium phosphate buffer containing 0.1% mercaptoethanol in pre-chilled pestle and mortar, and 600 mesh carborundum (Fisher Scientific, United States) was used as an abrasive. Indicator plants were pre-dusted with carborundum, and the sap was gently rubbed on to leaves. After 2 min of inoculation, the plants were washed with sterile water using a squeeze bottle and kept for observation in an insect-proof glass house. The virus inoculum was maintained under glasshouse conditions.

### 2.4 Collection of bacterial endophytes

The bacterial endophytes, viz., *Bacillus licheniformis* (KC540811), *Bacillus tequilensis* (MW301641), *Brachybacterium paraconglomeratum* (MK263736), *Myroides odoratimimus* (MWO82530), *Bacillus sonorensis* (MT331689), *Bacillus megaterium* (KC540802), *Bacillus velezensis* (MW331688), *Bacillus velezensis* (VB7) (KJ603234), *Stenotrophomonas maltophilia* (MN082440), *Bacillus subtilis* (KJ540802), and *Bacillus paralicheniformis* (MTW301648) were obtained from the Department of Plant Pathology, TNAU, Coimbatore, and another 10 bacterial endophytes, viz., *Bacillus boroniphilus* (MTCC9853), *Bacillus bataviensis* (MTCC7309), *Bacillus atrophaeus* (ABO21181), *Bacillus cereus* (KACC100001), *Bacillus azotoformans* (MTCC2598), *Bacillus cohnii* (MTCC3016), *Bacillus safensis* (AF234854), *Bacillus pumilus* (AY456263), *Bacillus circulans* (IAMI12462), and *Bacillus stratosphericus* (AJ831841) were obtained from the Department of Agricultural Microbiology, TNAU, Coimbatore. The pure culture of the bacterial endophytes was maintained at 28 ± 2°C in sterile Petri plate containing Luria-Bertani (LB) medium.

### 2.5 Screening for the antiviral activity of bacterial endophytes against GBNV in cowpea (VBN 3)

#### 2.5.1 Preparation of bacterial inoculum

Twenty-one bacterial endophytes were maintained by inoculating 24-h old culture into LB broth and incubated in an orbital shaker at 150 rpm at 28 ± 2°C for 48 h. The OD value of the bacterial antagonist was adjusted to 1.5 at A600 nm (10^8^ CFU/mL) in all the experiments.

#### 2.5.2 Assay for the antiviral activity in cowpea

The bacterial endophytes were screened in cowpea seedlings (VBN3) to test their efficacy against GBNV. The virus isolate Deto (OR159681) from Devarayapuram location was used throughout the study for screening. Two treatments were performed with pre inoculation (the bacterial inoculum was sprayed with atomizer 24 h before inoculation of virus) and simultaneous inoculation (co-inoculation of virus and bacterial isolates) at 1.5% concentration (volume by volume) of crude culture of endophytes. GBNV was inoculated as per the standard procedure (Hull, 2009; Vinodkumar et al., 2018), and the virus concentration was maintained uniformly at 1.40 OD (A405 nm). The plants were maintained in insect-proof cages under glasshouse condition. Periodical observations were recorded on symptom expression. Disease reduction and inhibition over control were calculated based on lesion number per leaf. For each treatment, three replications and five plants per replication were maintained including healthy plants. The results of preliminary experiments revealed that simultaneous inoculation performed well in reducing the lesion compared with pre inoculation. Therefore, for further assessment of virus mitigation studies, the simultaneous inoculation method alone was employed. The percentage of reduction over control was calculated by using the formula as follows:


$$\begin{array}{l} \mathrm{Percent}\;\mathrm{reduction}\;\mathrm{over}\;\mathrm{control}=\\ \;\frac{\mathrm{Number}\;\mathrm{of}\;\mathrm{lesions}\;\mathrm{in}\;\mathrm{control}-\mathrm{Number}\;\mathrm{of}\;\mathrm{lesions}\;\mathrm{in}\;\mathrm{treatments}}{\mathrm{Number}\;\mathrm{of}\;\mathrm{lesions}\;\mathrm{in}\;\mathrm{control}} \end{array}$$

### 2.6 Quantification of GBNV titer in cowpea

#### 2.6.1 DAC-ELISA

The virus titer in the bacterial endophyte-treated cowpea plants was assessed by Direct Antigen Coating Enzyme-linked Immunosorbent Assay (DAC-ELISA) using polyclonal antisera of GBNV obtained from ICRISAT, Hyderabad, India, according to the procedure by Hobbs et al. (1987). Samples were collected 4 days after challenge inoculation with GBNV. Dilution of 1:10000 was used for primary antibody, and anti-rabbit IgG (Cat No.#1100180011730, Sigma, Germany) was used as secondary antibody with the dilution of 1:5000. The readings were recorded after 30 min of incubation. The experiment was performed with three biological replications for each treatment with two technical replications. Samples with double fold absorbance at 405 nm than the healthy control were considered as positive (Clark and Adams, 1977).

#### 2.6.2 Real-time PCR

Quantification of the virus was assessed by using GBNV nucleocapsid primer (GBNV F-5′GGACCAGATGACTGGACCTTC, GBNV R-5′TCGAAAGCTGCA GGGACAT T3′) (Vanthana et al., 2022), to amplify 167 bp through real-time PCR in the BIO-RAD CFX96 manager. Samples were drawn at different time intervals (24 h, 48 h, 72 h, and 96 h) upon simultaneous inoculation of bioagents and GBNV inoculum. Overall, 10 μL of reaction mixture contains 5 μL of SYBR Green master mix (KAPA SYBR @ FAST for Light Cycler 480, Cat. No. A1250), 10 pm/μL concentration of forward and reverse primers, 2 μL of nuclease-free water, and 1 μL of template cDNA with an amplification cycle of 95°C for 10 min (initial denaturation) and 40 cycles of 95°C for 30 s, 60°C for 30 s, and 72°C for 30 s, followed by standard melting temperature analysis. The viral copy number was quantified by the absolute quantification method using recombinant plasmid DNA containing the GBNV-CP gene in the pGEMT vector. The copy number was calculated by using the formula:

Y molecules = X g/ mL DNA × 6.022 × 10^23^/(Base pair of recombinant plasmid × 660)

### 2.7 Screening the antiviral efficacy of selected bacterial endophytes against GBNV in tomato

Based on initial screening, the effective bacterial endophytes, viz., *B. licheniformis* Soya 1, *B. velezensis* VB7, and *B. tequilensis* (NBL6) were selected to test their efficacy against GBNV in tomato (variety—Shivam) upon simultaneous inoculation (co-inoculation of virus and bacterial isolates) at 1.5% concentration of crude culture broth. The plants were incubated in insect-proof chamber for observation. The number of plants exhibiting symptoms, days taken for symptom expression, and percent reduction in disease incidence and systemic infection was recorded. Five replications were maintained with five plants per replications. Furthermore, the symptom severity was assessed in 0 to 4 grades (where 0—no symptom, 1—mild symptom, 2—moderate symptom, 3—severe symptom, and 4—severe symptom with stem necrosis) (Vanthana et al., 2019). The percentage of disease index was calculated and correlated with the virus load following the procedure outlined by Vanthana et al. (2019). DAC ELISA was performed to compare the virus titer in tomato plants treated with bacteria challenged with GBNV, untreated virus inoculated, and uninoculated plants in tomato. Samples were collected at different days after inoculation (0, 5, and10 DPI) of the virus. The experiment was conducted with three representative leaf samples per treatment with two technical replications along with healthy and buffer control. Furthermore, absolute quantification of the virus was accomplished through real-time PCR.

### 2.8 Statistical analysis

All the experiments were performed in triplicates, and the mean values were compared with ANOVA using Duncan’s multiple range test at 5% level of significance. All data were statistically analyzed and interpreted using IBM SPSS windows version 27. The principal component analysis was performed in R studio 4.2.0.

## 3 Results

### 3.1 Collection of GBNV-infected tomato samples

**Figure 1:**
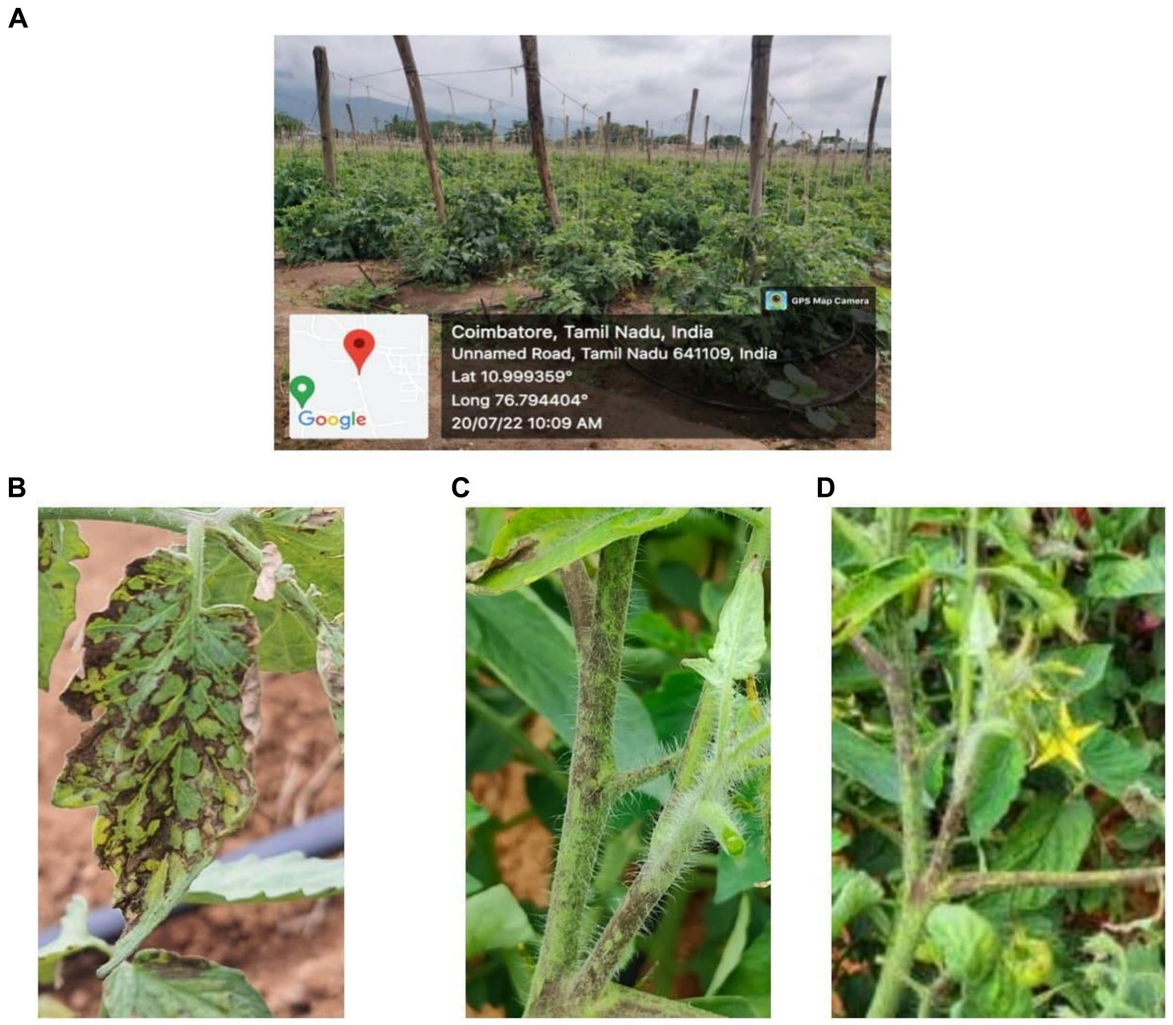
Symptom observed in the field. **(A)** GPS data-Devarayapuram, Coimbatore. **(B)** Necrosis on leaf. **(C)** Necrosis on stem. **(D)** Necrosis on petiole.

Tomato samples infected with GBNV exhibiting necrotic ring spots on leaves and stem necrosis were collected from major tomato-growing tracts of Devarayapuram, Boluvampatti, Marchinaickenpalayam, and Tholampalayam of Coimbatore district and Hosur region of Krishnagiri district, Tamil Nadu province, India. Various symptoms of GBNV, including necrotic ring spots on leaves, necrosis on buds and necrotic streaks on the stem with stunted growth, and deformed leaves were observed in the tomato field (Figure 1).

### 3.2 Molecular characterization of GBNV-infecting tomato

**Figure 2:**
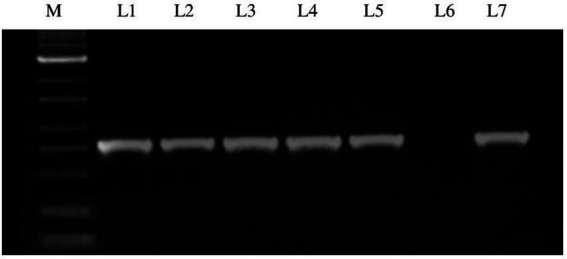
Agarose gel electrophoresis of RT-PCR product of GBNV nucleocapsid gene of infected tomato samples. Lane M: 1 kb ladder; Lane 1: Infected samples from Boluvampatti; Lane 2: Devarayapuram; Lane 3: Marchinaikenpalayam; Lane 4: Tholampalayam; Lane 5: Hosur; Lane 6: Negative control; Lane 7: Positive control.

The RNA extracted from the GBNV-infected samples was subjected to RT-PCR using specific primers of the nucleocapsid gene (N) of GBNV. The expected amplicon size of ~830 bp was obtained in the five samples collected from various locations of Coimbatore district and Krishnagiri district, which confirmed the presence of GBNV (Figure 2). The PCR products were sequenced, and edited final sequences of N gene of different isolates are available under the following accession number in NCBI databases. Accession numbers were obtained as OR158681 (Devarayapuram-DeTo), OQ473394 (Boluvampatti BoTo), OQ473392 (Marchinaickenpalayam MaTo), OQ473393 (Tholampalayam ThoTo), and OQ124156 (Hosur HoTo), which had 96–97% identity with GBNV isolate from Coimbatore (AY472081).

**Figure 3:**
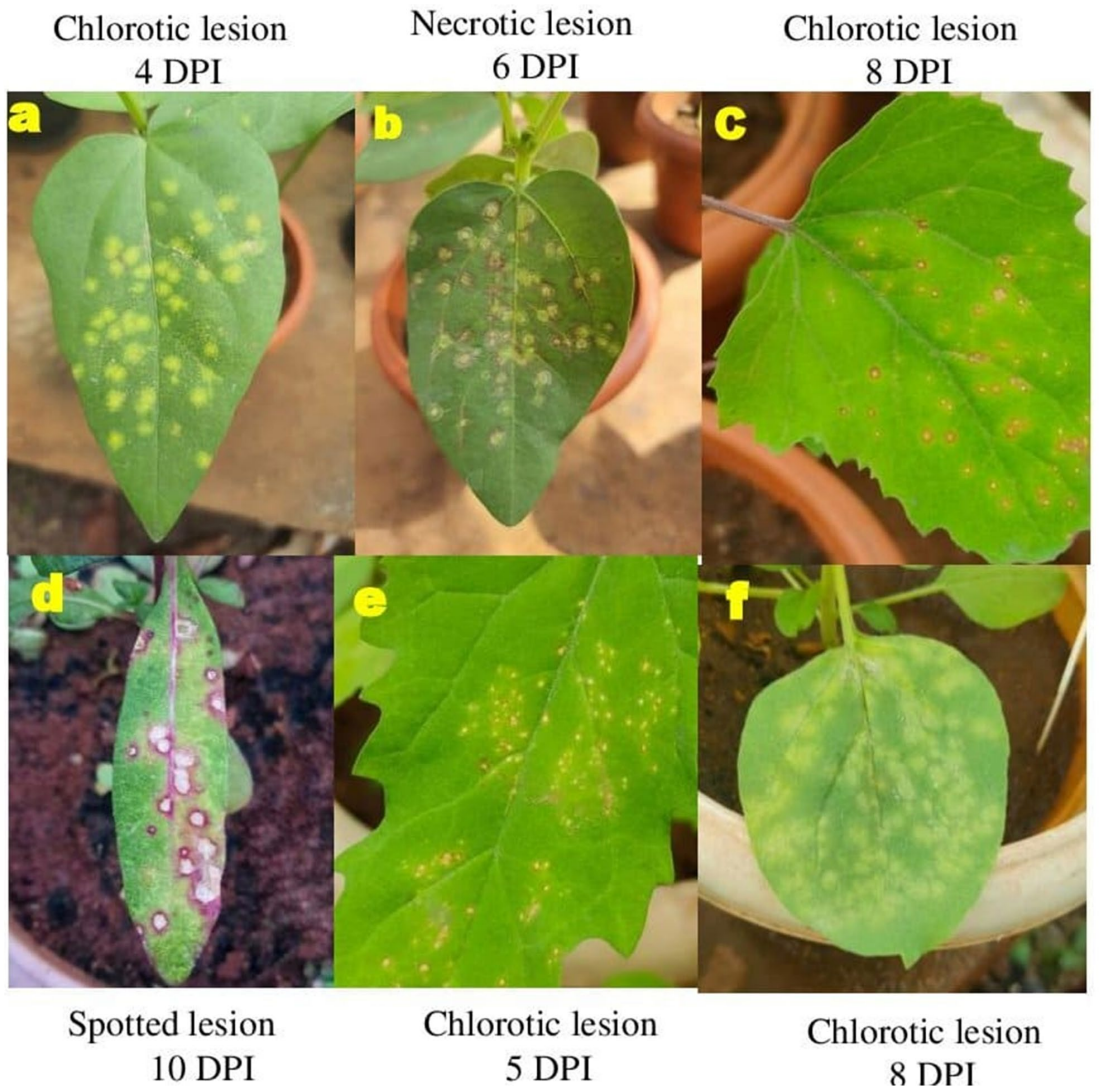
Symptom expression of GBNV in assay hosts. **(A,B)** Cowpea. **(C)** *Chenopodium amaranticolor*. **(D)** *Gomphrena globosa*. **(E)** *Chenopodium quinoa*. **(F)** *Nicotiana benthamiana*.

The GBNV inoculum of DeTo isolate was maintained in different assay hosts for further studies. Different types of symptoms were observed in all the hosts, and the time taken for symptom expression differed significantly. Among the various assay hosts, cowpea leaves expressed initially chlorotic lesions followed by necrotic lesions at the earliest of 4 DPI followed by *Nicotiana benthamiana*, *Chenopodium amaranticolor*, and *Chenopodium quinoa*, which expressed the necrotic lesions at 8 DPI. However, *Gomphrena globosa* exhibited initially chlorotic lesion which later turned as necrotic lesions at 10 DPI (Figure 3).

### 3.3 Screening of bacterial antagonists for antiviral efficacy against GBNV

**Figure 4:**
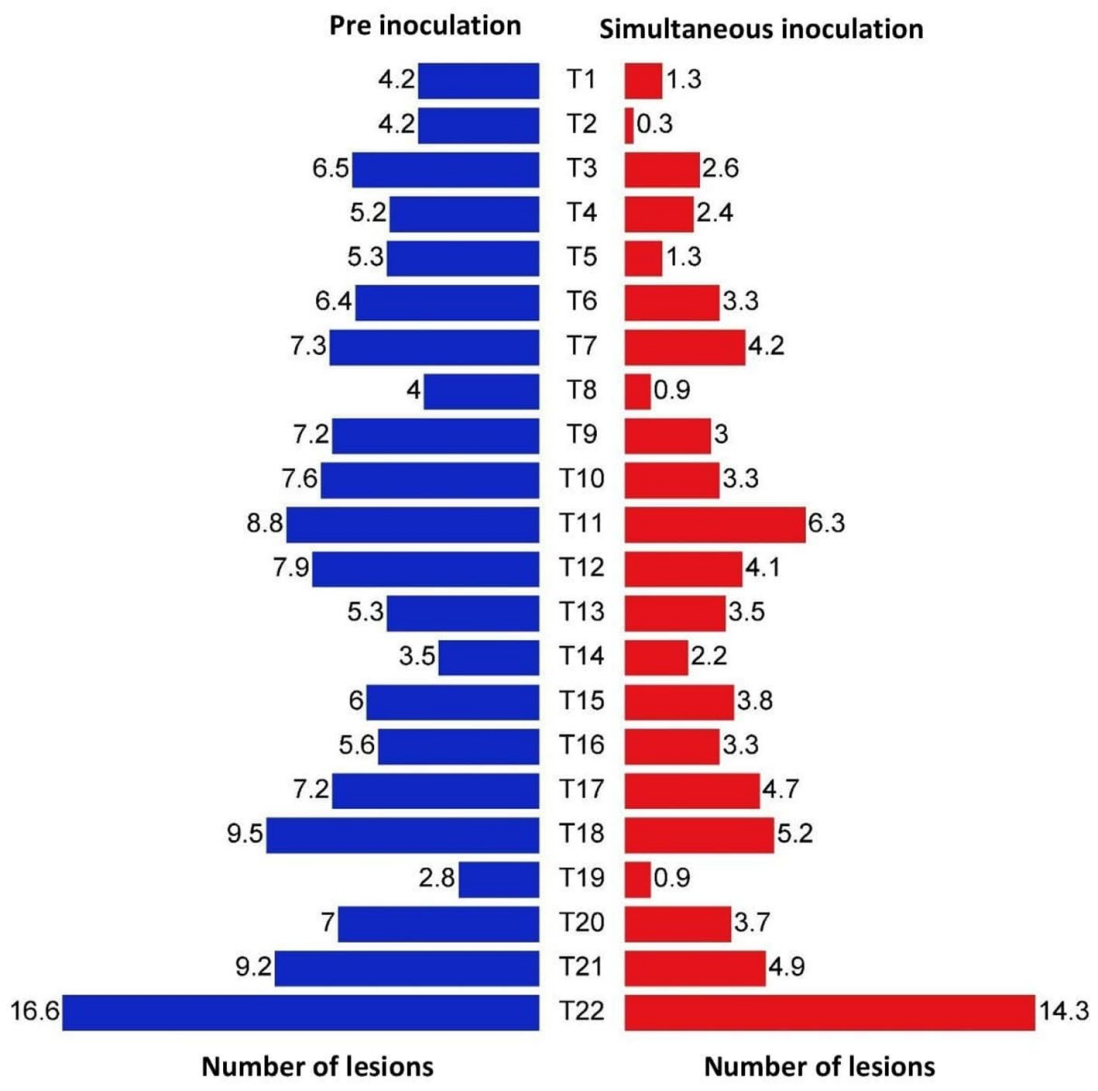
Efficacy of bacterial endophytes in the reduction of lesion numbers in cowpea (VBN3) upon pre and simultaneous inoculation with GBNV. T1-Soya 1; T2-NBL6; T3-YEB PT2; T4-YEB RT3; T5-KMR3; T6-BAG 3; T7-PL7; T8-VB7; T9-YEB RH2; T10-VB9; T11-ASD 16S1; T12-MTCC 9853; T13-MTCC 7309; T14-JCM9080; T15-KACC 100001; T16-MTCC 2598; T17-MTCC 3616; T18-F0-036; T19-DSM 27; T20-IAMI 12462T21-41KF2a; T22-Untreated inoculated control.

In both simultaneous and pre inoculation treatments, all the inoculated leaves expressed typical chlorotic yellow spots on 4 DPI which later turned into necrotic lesions. However, there was a significant difference in progress of the disease in treated plants. Simultaneous inoculation of GBNV and bacterial endophytes in cowpea was more effective compared with pre-inoculation treatment. Among the simultaneous spray treatments, *B. tequilensis* (NBL6) was effective in reducing the number of lesions to 0.33 that was reduced by 98.01% over inoculated control followed by *B. velezensis* (VB7), which inhibited GBNV lesions upto 94.70% over control. *B. licheniformis* (Soya 1) and *B. sonorensis* (KMR3) were equally effective in reducing the lesions to 1.25 which accounted for 92.10% reduction over control. The lowest inhibition of 62.05% was observed in *B. paralicheniformis* (ASD16S1)*-*treated plants. The uninoculated plants exhibited the maximum number of lesions (14.33) compared with bacterized leaves. In pre-inoculation treatments, 16.60 lesions were recorded in untreated inoculated control, whereas the plants treated with *B. velezensis* (VB7) had the least number of lesions (4.0) which was 75.90% reduction over control. It was followed by 4.17 lesions in *B. licheniformis* (74.88%) and 4.23 lesions in *B. tequilensis* (74.52%) (Table 1 and Figure 4; Supplementary Figures S1, S2).

**Table 1:** Efficacy of bacterial endophytes against GBNV in cowpea (VBN 3) upon pre and simultaneous inoculation.

S. No.	Treatments (crude culture suspension)	Total number of lesions/two leaf^a^			
		Pre inoculation spray	Percent reduction over control	Simultaneous inoculation spray	Percent reduction over control
1	*Bacillus licheniformis* (Soya 1)	4.17 (11.77)	74.88	1.30 (6.54)	92.17
2	*Bacillus tequilensis* (NBL6)	4.23 (11.85)	74.52	0.33 (3.29)	98.01
3	*Brachybacterium paraconglomeratum* (YEB PT2)	6.50 (14.53)	60.84	2.61 (9.29)	84.28
4	*Myroides odorotimimus* (YEB RT3)	5.18 (13.10)	68.80	2.44 (8.98)	85.30
5	*Bacillus sonorensis* (KMR3)	5.33 (13.34)	67.89	1.25 (6.41)	92.47
6	*Bacillus megatherium* (BAG 3)	6.40 (14.64)	61.45	3.27 (10.43)	80.30
7	*Bacillus velezensis* (PL7)	7.27 (15.63)	56.20	4.20 (11.81)	74.70
8	*Bacillus velezensis* (VB7)	4.00 (11.53)	75.90	0.88 (5.38)	94.70
9	*Stenotrophomonas maltophilia* (YEB RH2)	7.21 (15.56)	56.57	3.00 (9.96)	81.93
10	*Bacillus subtilis* (VB9)	7.61 (16.00)	54.16	3.33 (10.51)	79.94
11	*Bacillus paralicheniformis* (ASD 16S1)	8.77 (17.22)	47.17	6.30 (14.60)	62.05
12	*Bacillus boroniphilus* (MTCC 9853)	7.87 (16.28)	52.59	4.10 (11.67)	75.30
13	*Bacillus bataviensis* (MTCC 7309)	5.33 (13.34)	67.89	3.5 (10.78)	78.92
14	*Bacillus atrophaeus* (JCM 9070)	3.47 (10.73)	79.10	2.2 (8.51)	86.75
15	*Bacillus cereus* (KACC 100001)	6.00 (14.17)	63.86	3.8 (11.23)	77.11
16	*Bacillus azotoformans* (MTCC 2598)	5.63 (13.72)	66.08	3.3 (10.46)	80.12
17	*Bacillus cohnii* (MTCC 3616)	7.17 (15.52)	56.81	4.7 (12.51)	71.69
18	*Bacillus safensis* (F0-036)	9.54 (17.98)	42.53	5.2 (13.17)	68.67
19	*Bacillus pumilus* (DSM 27)	2.77 (9.57)	83.31	0.9 (5.44)	94.58
20	*Bacillus circulans* (IAMI 12462)	7.00 (15.33)	57.83	3.7 (11.08)	77.71
21	*Bacillus stratosphericus* (41KF2A)	9.23 (17.67)	44.40	4.9 (12.78)	70.48
22	Inoculated control	16.60 (23.03)	—	14.33 (22.15)	
23	Uninoculated control	—		—	
	CD	0.427		0.583	
	SE (d)	0.211		0.280	

Values in the parentheses are arcsine transformed values.

a Mean value represents three replications, and each replication contains five plants.

### 3.4 Assessment of virus titer by DAC ELISA

**Figure 5:**
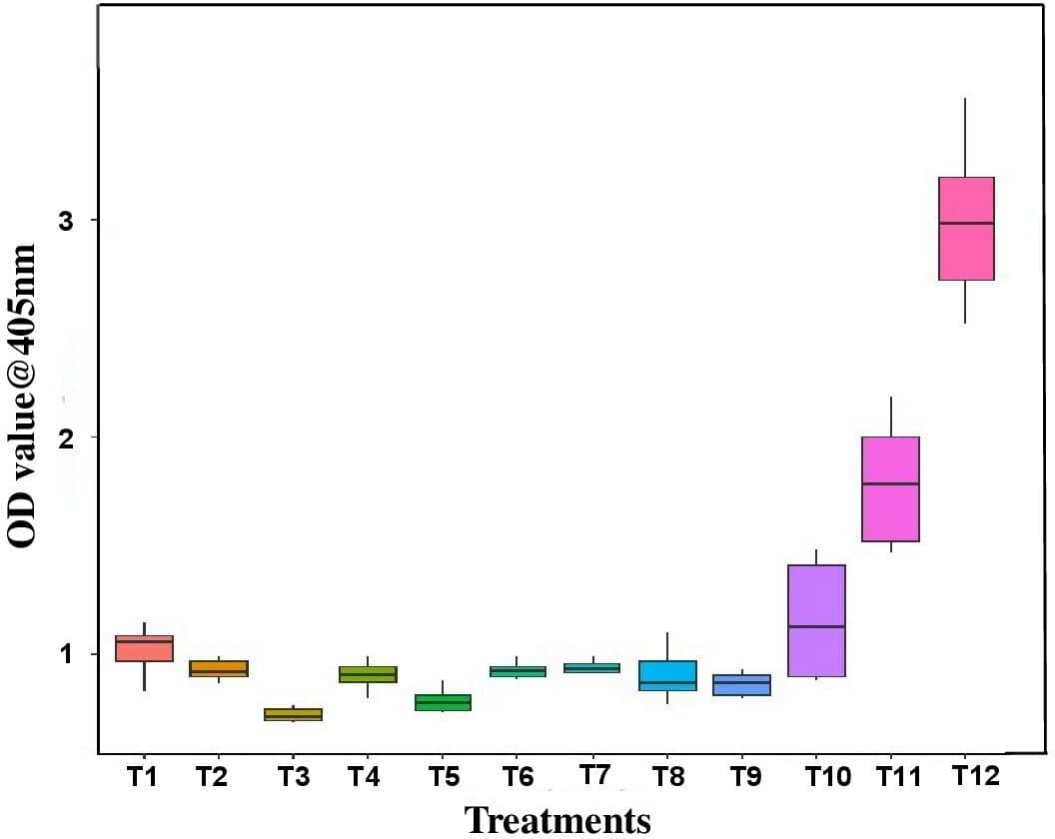
Assessment of GBNV titer in bioagent-treated cowpea (VBN3) through DAC ELISA. T1-YEB RT3; T2-KMR3; T3-VB7; T4-Soya 1; T5-NBL6; T6-DSM27; T7-MTCC 2598; T8-JCM 9080; T9-MTCC 7309; T10-IAMI12462; T11-Positive control; T12-Untreated inoculated control.

Out of 21 bacterial endophytes screened, 10 effective endophytes were selected based on pre and simultaneous inoculation studies. The virus titer due to antiviral activity of endophytes was assessed through DAC ELISA. The effective bacteria selected include *B. licheniformis*, *S. maltophila*, *B. velezensis*, *B. sonorensis*, *M. odoratimimus*, *B. azotoformans*, *B. atrophaeus*, *B. bataviensis*, *B. circulans*, and *B. pumilus*. The results revealed that the inoculated untreated plants had the highest OD value of 2.993 at A405 nm, whereas the bacterial endophyte-treated plants had very less OD value of 0.661 in *B. tequilensis* (NBL6)*-*treated plants followed by 0.773 in *B. velezensis* (VB7)-treated plants. In healthy control, the OD value at A405 nm was 0.634. Thus, the results confirmed that the bacterial endophyte treatments reduced the GBNV titer in cowpea (Figure 5).

### 3.5 Assessment of virus titer by qPCR

Based on the previous experiment, the five effective bacterial endophyte treatments (*B. velezensis* (VB7), *B. licheniformis* (Soya 1), *B. tequilensis* (NBL9), *B. sonorensis* (KMR3), and *M. odorotimimus* (YEBRT3)) were selected for qPCR study. qPCR was performed using GBNV nucleocapsid primer (GBNV F-5′ GGACCAG ATGACTGGACCTTC, GBNV R-5′TCGAAAGCTGCAGGGACAT T3′) (Vanthana et al., 2019). Cowpea leaf samples were collected at different time intervals (0 h, 24 h, 48 h, 72 h, and 96 h) upon simultaneous inoculation of bioagents and GBNV inoculum. In real-time PCR, a gradual increase in virus copy number was noticed in all the treatments from 0 h to 96 h after inoculation. On 96 h of inoculation, the virus copy number was highest in the case of inoculated control (1.2 × 10^8^). The virus copy numbers were reduced in bio-agent-treated cowpea plants. The virus copy number was 2.4 × 10^7^ copies in *B. licheniformis* Soya 1-treated plants, 3.5 × 10^6^ in *B. velezensis* VB7, and 3.6 × 10^6^ in *B. tequilensis* NBL6 when compared with 1.2 × 10^8^ copies in inoculated control after 96 h of inoculation (Supplementary Figure S3 and Supplementary Table S2).

### 3.6 Screening of effective bacterial endophytes against GBNV in tomato

**Figure 6:**
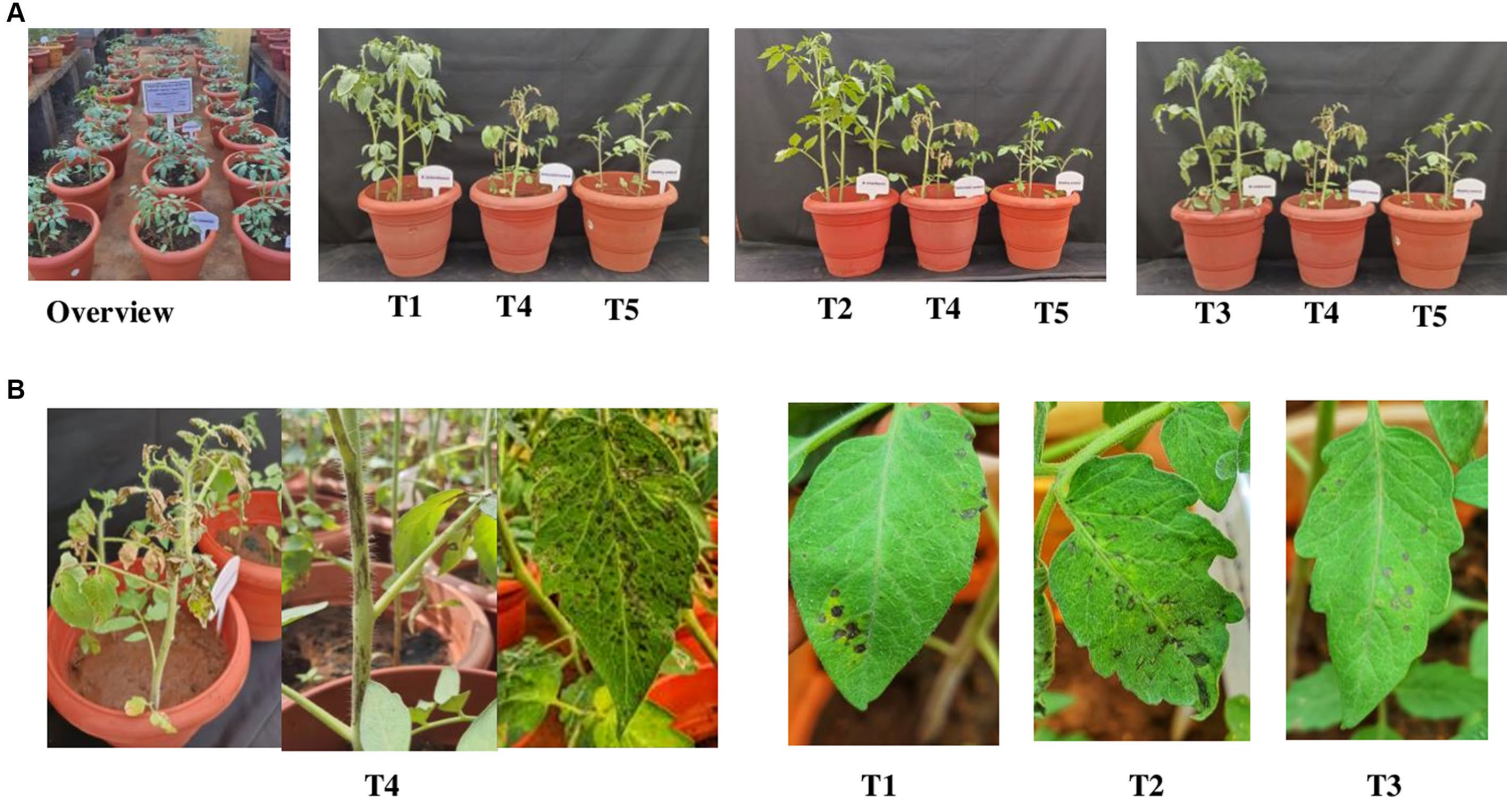
**(A)** Efficacy of bacterial endophytes against GBNV in tomato. **(B)** Symptom expression in treatments. T1-*B. velezensis* (VB7), T2-*B. licheniformis* (Soya 1), T3*-B. tequilensis* (NBL6), T4-Untreated inoculated control, T5-Healthy control.

Among, the 3 bacterial endophytes (*B. licheniformis* Soya 1, *B. tequilensis* NBL6, and *B. velezensis* VB7) screened, *B. licheniformis* Soya 1 and *B. tequilensis-*treated plants had 16% disease incidence followed by *B. velezensis* VB7 which had 24% disease incidence. However, the maximum incidence of 88% was recorded in untreated inoculated control. Furthermore, the delay in symptom expression was recorded in bacterial endophyte-treated plants. In untreated inoculated plants, the symptom was expressed within 10 days, whereas, in the treated plants, symptom expression was delayed to 13 days in *B. velezensis* VB7 and 12 days in *B. tequilensis* NBL6 followed by *B. licheniformis* Soya 1*-*treated plants (11 days) with mild symptoms (Figures 6A,B). The endophyte-treated plants exhibited vigorous growth than the healthy untreated plants. Furthermore, the systemic infection of virus symptoms was observed in untreated inoculated plants, which resulted in complete drying of the whole plants after 15 DPI. Inoculation with GBNV resulted in 90% infection in virus-inoculated plants. These plants exhibited a symptom severity grade of 3.08 with 77 percent disease index (PDI), which had the characteristic complete drying of leaves and veinal necrosis symptoms. Contrastingly, the symptom severity grade in *B. tequilensis* (NBL 6)-treated plants was 0.42 (10 PDI) followed by 0.50 (12.5 PDI) severity grade in *B. licheniformis* Soya 1-treated plants (Table 2).

**Table 2:** Assessment of percent disease incidence of GBNV in various treatments.

Treatment	Number of symptomatic plants/total number of plants inoculated						% Disease incidence (DPI)					Disease reaction	Severity index
	*R*_1_	*R*_2_	*R*_3_	*R*_4_	*R*_5_	Total infected plants	3	6	9	12	Mean		
*B. velezensis* (VB7)	1/5	0/5	0/5	2/5	1/5	4	0	0	0	16	16	Highly tolerant	0.64
*B. licheniformis* (Soya 1)	2/5	1/5	1/5	1/5	1/5	6	0	0	0	24	24	Tolerant	0.50
*B. tequilensis* (NBL6)	1/5	0/5	0/5	3/5	0/5	4	0	0	0	16	16	Highly tolerant	0.42
Untreated inoculated control	4/5	5/5	5/5	4/5	5/5	22	0	0	60	28	88	Susceptible	3.08
Healthy control	0/5	0/5	0/5	0/5	0/5	0	0	0	0	0	0	—	0

### 3.7 Assessment of GBNV titer by DAC ELISA in endophyte-treated tomato plants

**Figure 7:**
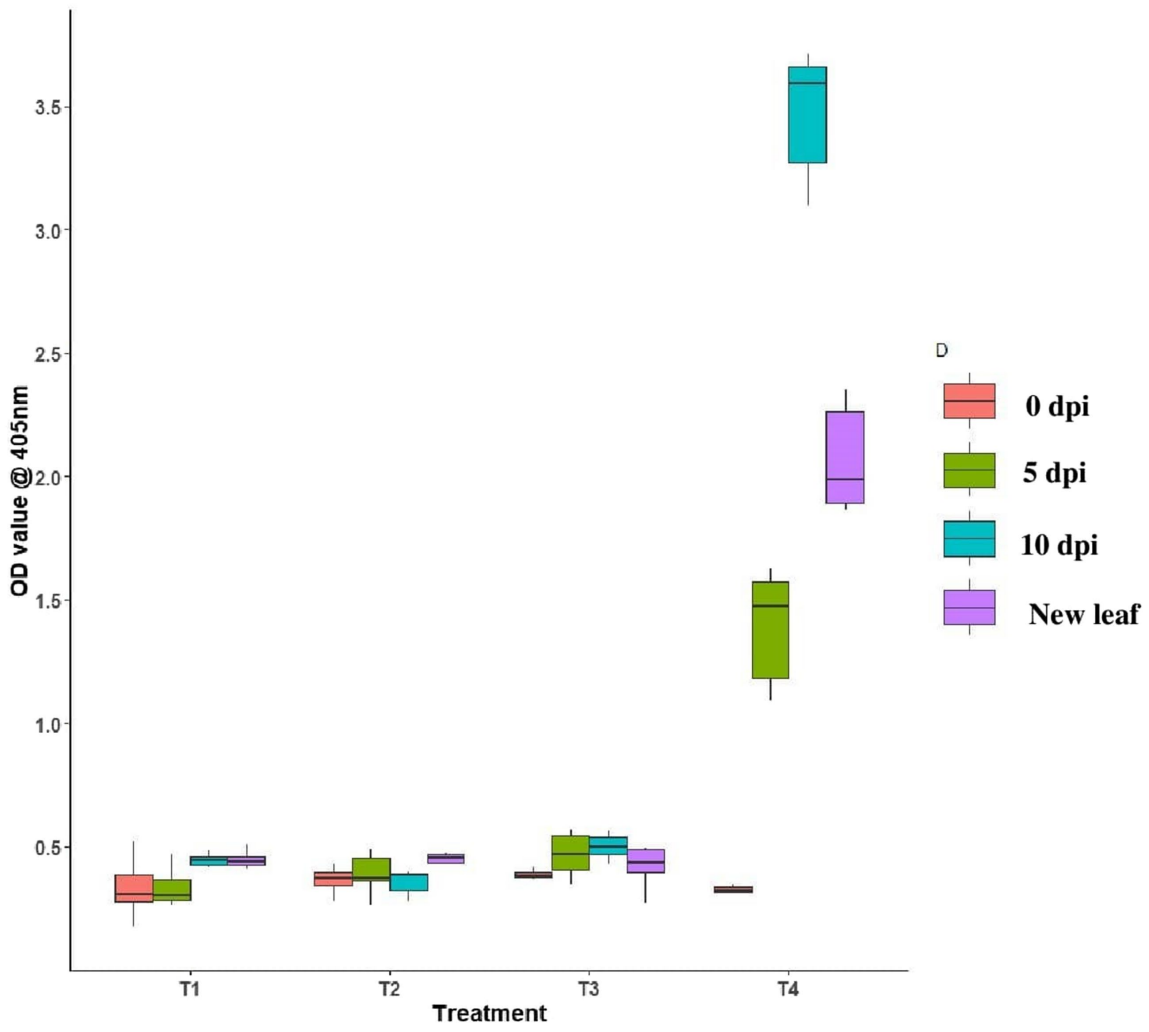
Assessment of GBNV in endophyte-treated tomato plants through DAC ELISA. T1-*B. velezensis* (VB7), T2-*B. licheniformis* (Soya 1), T3*-B. tequilensis* (NBL6), T4-Untreated inoculated control.

Tomato leaves bacterized with *B. tequilensis* had the least OD value of 0.382, 0.389, and 0.396 at 0th day, 5th day, and 10th DPI inoculation, respectively. In the case of newly emerged leaves, the OD value was least in *B. licheniformis* Soya 1*-*treated plants (0.398), whereas the OD value was 0.382, 0.284, and 0.430 at 0th, 5th, and 10th DPI against untreated inoculated control with OD value of 0.316, 1.392, and 3.475, respectively. In the case of untreated inoculated control plants, the newly emerged leaves had an OD value of 2.068. OD value of all the three endophyte-treated leaves was comparatively lesser on both 5th and 10th day over untreated inoculated control, which indicated that all three endophytic bacteria were effective in reducing the virus titer in treated plants (Figure 7).

### 3.8 Correlation of symptom severity in relation to virus titer in tomato plants bacterized with endophytes

**Figure 8:**
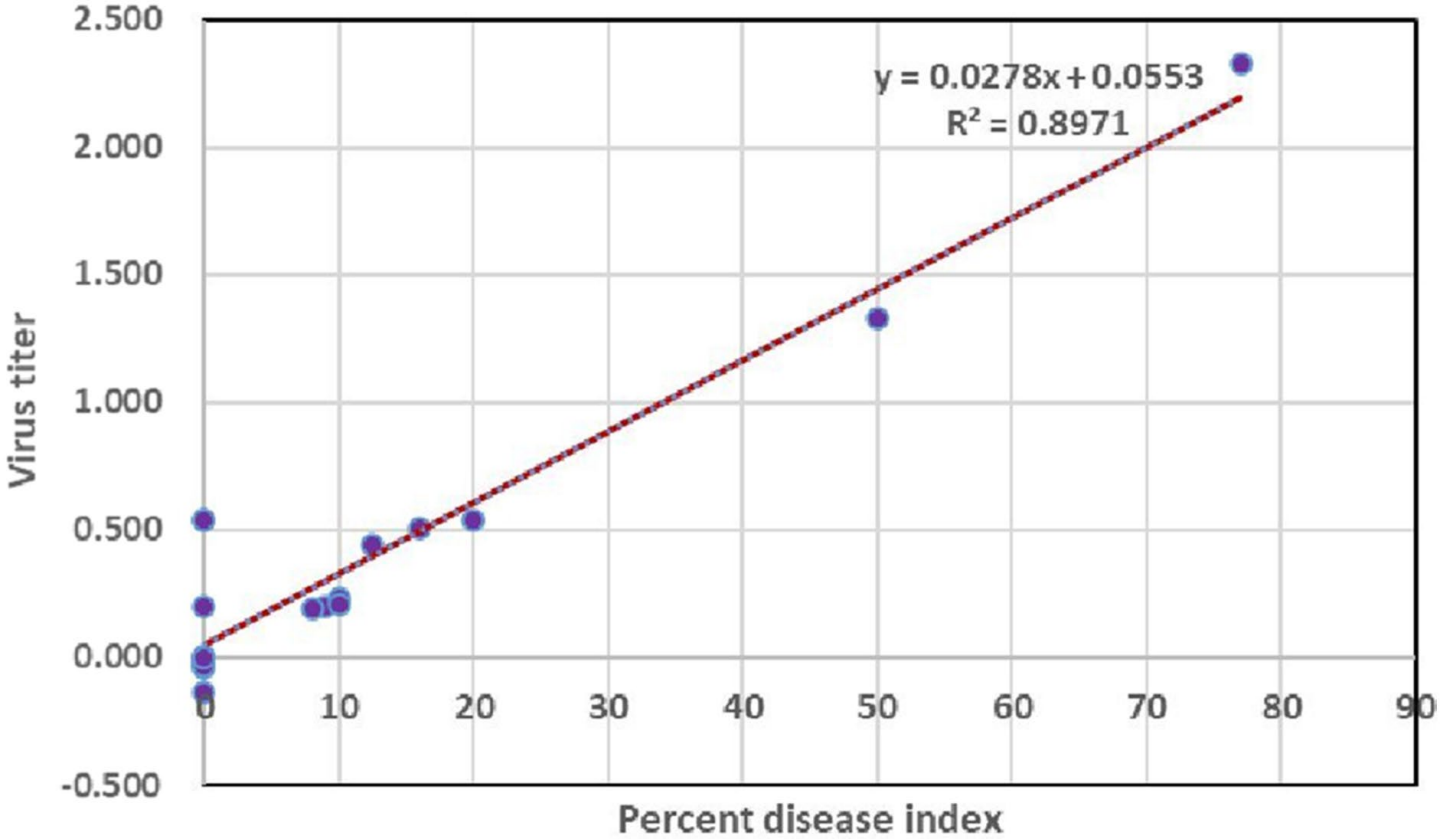
Assessing the correlation of virus titer with symptom severity.

To understand whether there is any significance between the percent disease index and concentration of the virus, a linear regression analysis was performed using a percent disease index as a dependent variable and virus load as an independent variable for various days after inoculation (3, 5, 7, 9, and 10 DPI). The *R*^2^ value of the regression analysis was 0.89 which indicated that the virus titer had an influence on percent disease index up to 89%. Furthermore, the *p*-value of 0.0002 denoted the significance between percent disease index and concentration of the virus titer at 1% level (Figure 8).

### 3.9 Absolute quantification of GBNV

The virus copy number was calculated at different days of virus inoculation on tomato (3rd, 5th, 7th, 9th, and 11th DPI). The virus load increased consistently from 3rd day to 11th day after inoculation of virus in both bacterial endophytes and untreated tomato plants challenged with GBNV, but significant reduction of GBNV load in the bacterial endophyte-treated plants was observed in comparison with untreated inoculated control from 5 DPI. The copy numbers were highest as 3.2 × 10^8^ in untreated inoculated control. However, the least copy number of 10^6^ fold was observed in *B. tequilensis* which was on par with *B. velezensis* (VB7)-treated plants. However, the copy number was 1.4 × 10^7^ in *B. licheniformis*-treated plants at 12 DPI (Table 3).

**Table 3:** Assessment of GBNV nucleocapsid gene copy number in bioagent-treated tomato (variety Shivam) through real-time PCR.

Treatment	3rd DPI	5th DPI	7th DPI	9th DPI	12th DPI
*B. velezensis* (VB7)	3.6 × 10^6^	3.8 × 10^6^	4.2 × 10^6^	4.5 × 10^6^	4.9 × 10^6^
*B. licheniformis* (Soya 1)	3.9 × 10^6^	3.9 × 10^6^	4.2 × 10^6^	4.3 × 10^6^	1.4 × 10^7^
*B. tequilensis* (NBL6)	3.7 × 10^6^	4.2 × 10^6^	4.5 × 10^6^	5.0 × 10^6^	5.0 × 10^6^
Untreated inoculated control	2.6 × 10^7^	1.5 × 10^7^	6.8 × 10^7^	7.6 × 10^7^	3.2 × 10^8^

### 3.10 Principal component biplot analysis of virus titer and disease incidence in response to bacterization of tomato leaves with different bacterial antagonists

**Figure 9:**
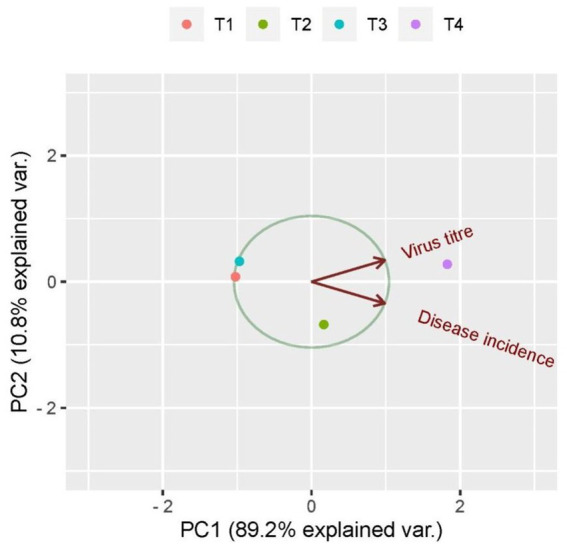
Principal component analysis correlating virus titer and disease incidence in response to bacterization of tomato leaves with different bacterial antagonists. T1-*B. velezensis* (VB7), T2-*B. licheniformis* (Soya 1), T3*-B. tequilensis* (NBL6), T4-Untreated inoculated control.

The principal component biplot depicts the correlation between treatments with the virus load and disease incidence. First, principal component accounted for 89.1% of the total variation among the treatments which included the virus titer and percent incidence. Among all the treatments, untreated inoculated control was loaded more on PC1 and created more variation. The treatments to the right side of the figure tend to have the least virus load and less expression of symptoms, whereas treatments to the left of the figure tend to have maximum viral load and was considered to express more disease incidence. The plants treated with *B. velezensis* VB7 and *B. tequilensis* gave similar scores. Hence, the tomato plants subjected to these treatments were considered as more tolerant to the virus infection compared with untreated inoculated control (Figure 9).

## 4 Discussion

Bud blight being a detrimental disease in tomato can cause the yield losses up to 100% depending on the stage of infection (Venkata Ramana et al., 2011). So far, we rely on pesticide for managing viral epidemics. Considering public health and environmental hazards, biological control has emerged as a sustainable alternative strategy for managing plant viral disease. Endophytic plant growth-promoting bacteria have been utilized for years as a means to promote plant growth and for the management of diseases caused by fungal and bacterial pathogens. However, exploiting their potential against plant viruses is only in the stage of infancy. Earlier research findings have demonstrated that the application of endophytic bacteria triggered the systemic defense mechanism against a variety of plant pathogens (Ryan et al., 2008). The application of *E. asburiae* significantly promoted plant growth and induced resistance against tomato yellow leaf curl virus upto 52% (Li et al., 2016). Under the experimental condition, the application of various bacterial endophytes viz., *Pseudomonas* spp., *Bacillus* spp., and *Burkholderia* spp., was well explored against plant viral diseases (Zehnder et al., 2000; Ramzan et al., 2016). With this knowledge, we assessed the antiviral efficacy of 21 bacterial endophytes against GBNV in local lesion host (cowpea) and tomato. Symptom expression and local lesion count were significantly reduced up to 90% by challenging GBNV with the endophytes viz., *B. tequilensis* (NBL6), *B. licheniformis* (Soya 1), and *B. velezensis* (VB7). Previous researchers have shown that the application of bacterial endophytes suppressed the viral infection in plants. The application of fluorescent *Pseudomonas* has reduced bhendi yellow vein mosaic incidence up to 86.67% (Patil et al., 2011), and the application of *B. subtilis* decreased the prevalence of PVY in potato (Amin et al., 2023) and PVX and PVY in tomato (Veselova et al., 2022). It was more evident in the case of *B. amyloliquefaciens* (VB7), as the present study agrees with the previous findings which demonstrated its antiviral activity and growth promotion against tobacco streak virus in cotton (Vinodkumar et al., 2018). In accordance with our results, Karthikeyan et al. (2024) reported that the application of *Bacillus* consortium before 24 h of CMV challenge effectively reduced the symptom expression and incidence of CMV in ridge gourd. The outcomes are in line with the findings by Shen et al. (2014) who reported that the application of *P. fluorescens* considerably reduced the infection of tobacco mosaic virus in tobacco plants up to 58.2% at field condition. The cucumber mosaic virus in pepper was effectively reduced by the application of *B. amyloliquefaciens* (Lee and Ryu, 2016). Gangireddygari et al. (2023) confirmed that the pepper mild mottle virus accumulation was markedly reduced to 43–47% in chilli plants treated with *P. putida* and *B. licheniformis* compared with control. Furthermore, maximum disease reduction of 84% of TSWV was observed in tomato plants treated with *P. fluorescens* (Kandan et al., 2005).

The virus titer was also reduced in the endophyte-treated cowpea and tomato plants challenged with GBNV. Compared with inoculated control (2.993 at A405 nm), the bacterial endophyte-treated plants had very less OD value of 0.661 in *B. tequilensis* (NBL6)*-*treated plants followed by 0.773 in *B. velezensis* (VB7)-treated plants against the healthy control (0.634). Earlier findings also emphasized that the application of bacterial endophytes reduced the virus titer. Ramzan et al. (2016) evaluated the application of *P. aeruginosa*, *Bacillus* spp., and *Burkholderia* sp. against cotton leaf curl virus in cotton of which the highest inhibition of cotton leaf curl virus titer was observed in combined application of endophytes (0.4%) as compared with 74% in untreated plants. Beris et al. (2018) demonstrated that tomato plants treated with *B. amyloliquefaciens* (MB1600) reduced the virus titer and enhanced the antiviral activity against TSWV and PVY. Similarly, various other studies have also emphasized that the application of *Bacillus* reduced the virus titer of tomato mosaic virus in tomato (Islam et al., 2016), cucumber mosaic virus in cucumber (Zehnder et al., 2000), and banana bunchy top virus in banana (Harish et al., 2009). It is notably promising that the application of bacterial endophytes exerts the delay in symptom manifestation that could range from 11 to 13 days depending on the species of bacterial endophytes. The delayed symptom expression in bacteria-treated plants may be attributed to an obstruction of virus movement or replication. However, Abdelkhalek et al. (2020) reported that the symptom expression of Alfalfa mosaic virus in potato was delayed by 3 days upon application of *B. licheniformis* before 24 h of inoculation of AMV. Analogously, the application of *Bacillus* delayed the GBNV infection in chilli (Rajamanickam and Nakkeeran, 2020). Similarly, the tomato yellow leaf curl infection was delayed by 5 days in *B. amyloliquefaciens*-treated plants and recorded mild infection than control (Guo et al., 2023).

Furthermore, we investigated systemic infection of virus in bacterial endophyte-treated plants. The results demonstrated the reduction in the virus titer in newly emerged leaves at 10 DPI. These results of preventing systemic infections are in line with previous studies which revealed that the foliar application of *Bacillus* spp. decreased systemic infection of potato virus Y (PVY) in potato after 10 and 16 days of inoculation with an inhibition of 66.74% over control (Amin et al., 2023). Similarly, Sorokan et al. (2020) reported that the application of endophytic bacteria in potato stopped the spread of potato virus Y during the initial phase of infection (7–14 DPI). Similar to our study, the viral copy number of GBNV was reduced in bioagent-treated plants compared with the untreated inoculated plants (Vanthana et al., 2022). Suppression of virus copy number was noticed up to 7.7 × 10^5^ copies in *B. velezensis* VB7 compared with control 1.01 × 10^8^ copies. Our findings corroborated with the results by El-Gendi et al. (2022) who reported that the foliar application of *B. subtilis* significantly reduced the disease severity and virus titer of tobacco mosaic virus in tomato and enhanced the growth and decreased virus accumulation in all treated tomato plants compared with non-treated plants.

In the present communication, we demonstrated the foliar application of *Bacillus* spp.-mediated disease reduction against GBNV in tomato. Lee and Ryu (2016) noted that the foliar application of biocontrol agents is not a common method compared with soil application. Many authors reported that the foliar application of bacterial culture filtrate is associated with delay in the development of plant virus-elicited symptoms (Abdelkhalek et al., 2020; Abo-Zaid et al., 2020). Consistent with our findings, the foliar application of *B. licheniformis* and *Streptomyces* sp. culture filtrate led to a noteworthy decrease in AMV and PVY accumulation in potato plants (Nasr-Eldin et al., 2019). Additionally, the application of *B. amyloliquefaciens* and *S. cellulosae* decreased the severity of TMV and CMV and the levels of viral accumulation in the treated leaves (Lee and Ryu, 2016; Abo-Zaid et al., 2020). Therefore, by preventing the accumulation of viral particles and triggering the plant defensive responses, the application of bacterial endophytes could shield tomato plants from GBNV infection.

In addition, the application of endophytes promoted the growth metrics of the plants. It has been reported that the application of *Bacillus* spp. can improve the plants via producing phytohormones (Karadeniz et al., 2006; Gamalero and Glick, 2011) and facilitates the uptake of insoluble phosphorus, iron, and trace elements (Meena et al., 2017). Islam et al. (2016) reported that the application of *Bacillus* spp. has promoted the germination percentage and seedling vigor and improved nitrogen content in cucumber plants. The application of bacterial endophytes displayed the hidden potential for inducing plant systemic resistance which may inhibit the virus infection through the production of phenolics and secondary metabolites including PR proteins (Manjunatha et al., 2022) and also the production of extracellular metabolites such as gibberellin, cytokinin, and IAA promoted the plant growth. The secondary metabolites of *Bacillus* spp. have both antimicrobial activity and can inhibit the growth of pathogen by decreasing iron content. Among the various inducers of resistance against plant pathogen, the application of *Bacillus* spp. attracts attention because of their advantages over other inducers, which include broad-spectrum antimicrobial activity and high levels of colonization on plant tissues with growth-promoting capacity (Shafi et al., 2017; Sansinenea, 2019).

The obtained results suggest that the studied *Bacillus* isolates not only promoted plant growth but also protected the plants from GBNV infection. Further research is needed to optimize the protocol for the administration of these *Bacillus* spp. and elucidate the molecular mechanisms involved in the resistance to GBNV. It is also hypothesized that the bacterial endophytes, when applied, may release MAMP molecules which may trigger PTI, the first line of defense. Whether *Bacillus* spp., interfere with the antiviral pathway of the hosts need to be investigated. Thus, the study clearly brought out the antiviral potential of bioagents and possibility in the management of devastating *Orthotospovirus* GBNV, which could lead to the development of virus mitigation strategy.

## Data availability statement

The original contributions presented in the study are included in the article/Supplementary material, further inquiries can be directed to the corresponding author’s.

## Author contributions

MG: Data curation, Investigation, Writing – original draft, Writing – review & editing. RS: Data curation, Validation, Writing – original draft, Writing – review & editing. PR: Conceptualization, Investigation, Methodology, Resources, Supervision, Writing – review & editing, Writing – original draft. SN: Conceptualization, Investigation, Methodology, Resources, Supervision, Writing – review & editing, Writing – original draft. NS: Software, Writing – original draft, Writing – review & editing. SV: Data curation, Visualization, Writing – original draft, Writing – review & editing. MR: Data curation, Visualization, Writing – original draft, Writing – review & editing. AS: Funding acquisition, Writing – original draft, Writing – review & editing. SA: Funding acquisition, Writing – original draft, Writing – review & editing.

## Footnotes

^1^ https://www.ncbi.nlm.nih.gov
