# Supplementary material for: Deciphering the antiviral nature of endophytic Bacillus spp. against groundnut bud necrosis virus in cowpea and tomato
Source: Front Microbiol. 2024 Jun 6;15:1410677. doi: 10.3389/fmicb.2024.1410677 (PMC11186990; doi:10.3389/fmicb.2024.1410677)
Supplement: Supplementary file 1 [file Table_1.DOCX]

Figure S1. Eﬃcacy of bacterial endophytes against GBNV in cowpea (VBN3) upon

pre inoculation


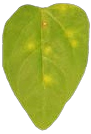

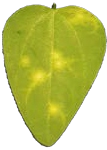

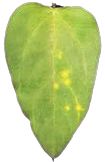

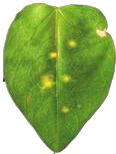

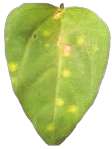

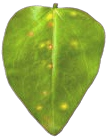

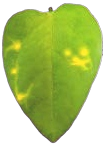

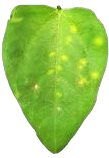

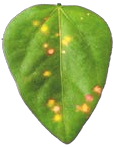

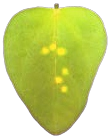

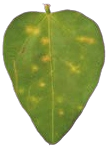

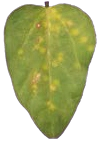

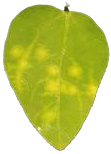

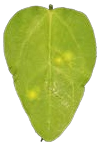

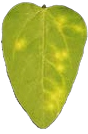

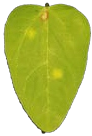

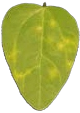

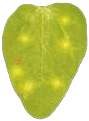

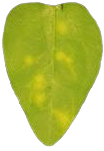

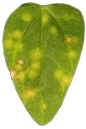

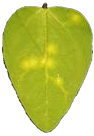

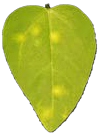

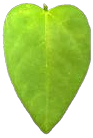


**T1**

**T2**

**T3**

**T4**

**T5**

**T6**

**T7**

**T8**

**T9**

**T10**

**T11**

**T12**

**T13**

**T14**

**T15**

**T16**

**T17**

**T18**

**T19**

**T20**

**T21**

**T22**

**T23**

**T1- Soya 1; T2- NBL6; T3- YEB PT2; T4-YEB RT3; T5-KMR3; T6-BAG 3; T7- PL7; T8- VB7; T9- YEB RH2; T10- VB9; T11- ASD 16S1*;* T12- MTCC 9853; T13- MTCC 7309; T14- JCM9080; T15- KACC 100001; T16- MTCC 2598; T17- MTCC 3616; T18- F0-036; T19- DSM 27; T20- IAMI 12462 T21- 41KF2a; T22- Untreated inoculated control; T23- Healthy control**

Figure S2. Eﬃcacy of bacterial endophytes against GBNV in cowpea (VBN3) upon

simultaneous inoculation


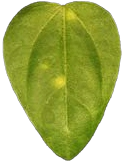

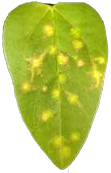

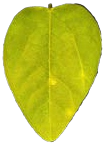

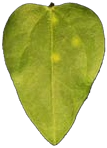

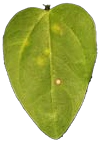

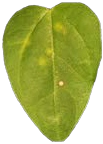

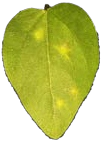

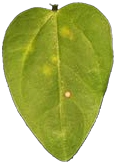

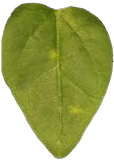

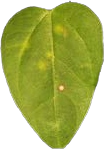

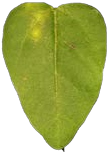

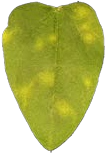

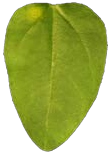

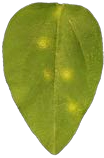

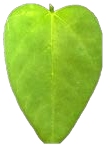

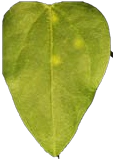

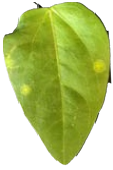

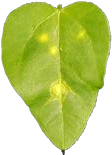

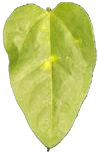

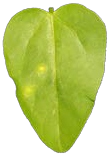

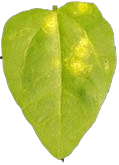

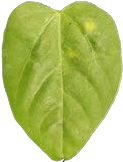

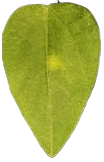


**T1**

**T2**

**T3**

**T4**

**T5**

**T6**

**T7**

**T8**

**T9**

**T10**

**T11**

**T12**

**T13**

**T14**

**T15**

**T16**

**T17**

**T18**

**T19**

**T20**

**T21**

**T22**

**T23**

**T1- Soya 1; T2- NBL6; T3- YEB PT2; T4-YEB RT3; T5-KMR3; T6-BAG 3; T7- PL7; T8- VB7; T9- YEB RH2; T10- VB9; T11- ASD 16S1*;* T12- MTCC 9853; T13- MTCC 7309; T14- JCM9080; T15- KACC 100001; T16- MTCC 2598; T17- MTCC 3616; T18- F0-036; T19- DSM 27; T20- IAMI 12462 T21- 41KF2a; T22- Untreated inoculated control; T23- Healthy control**


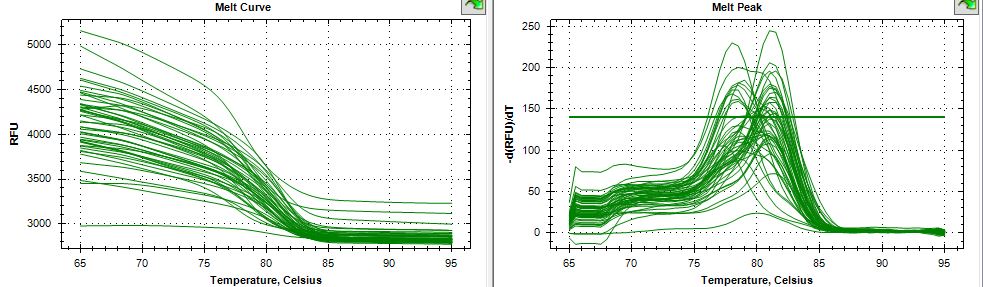

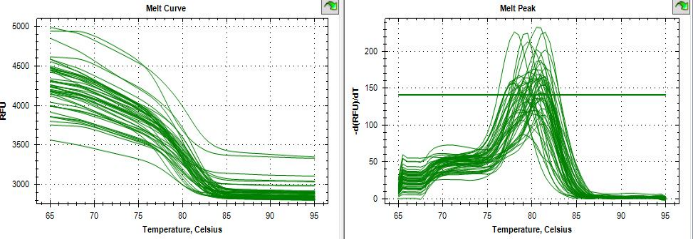

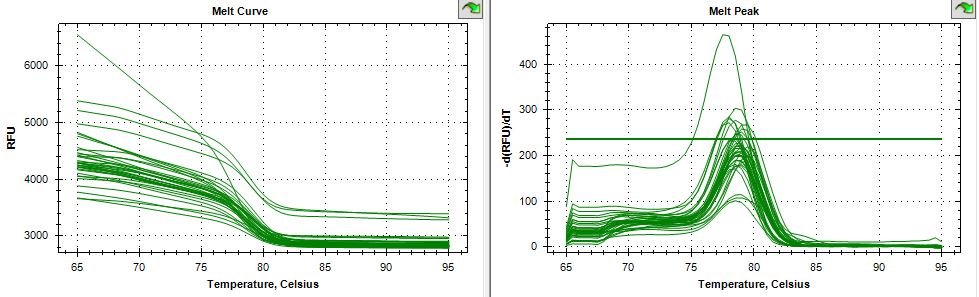

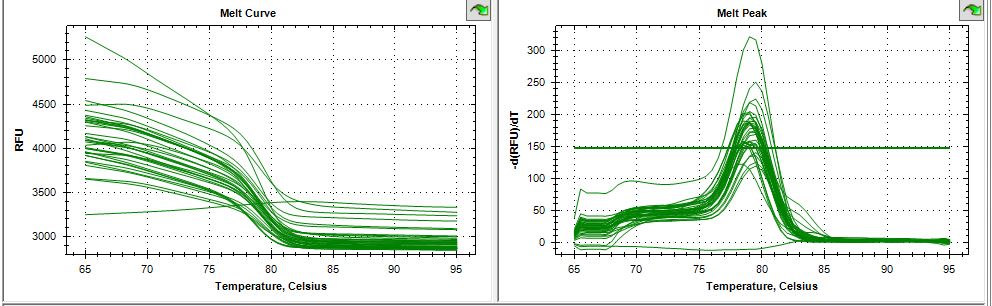

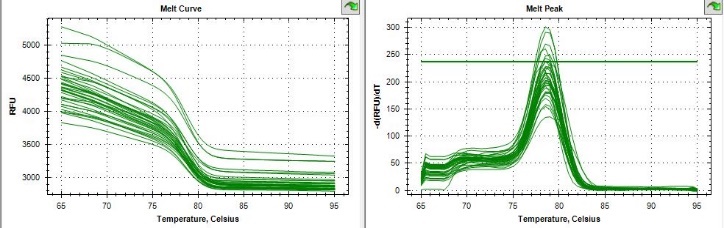


**96 h**

**48 h**

**24 h**

**12 h**

**0 h**

**Fig. S3. Melt peak and melt curves of GBNV nucleocapsid gene in qPCR assay in cowpea**

**Fig. S4. Symptom severity grade**


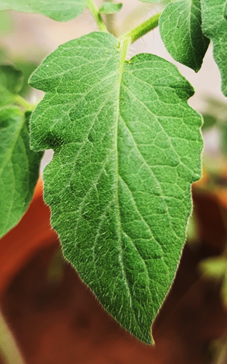

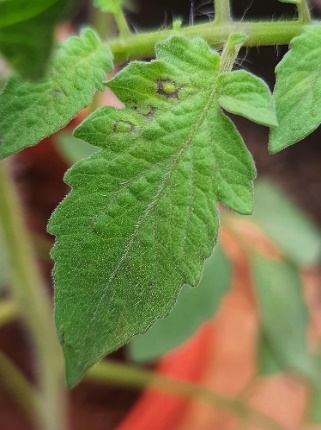

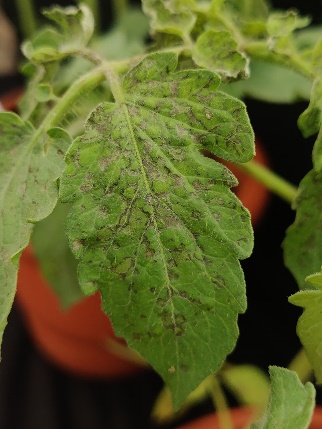


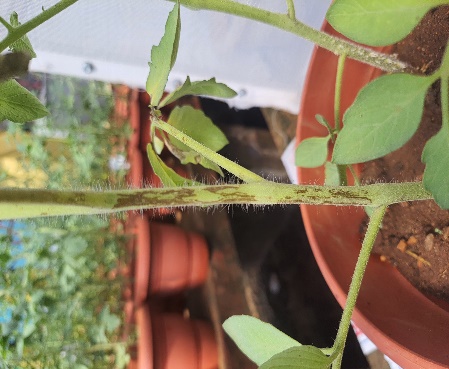

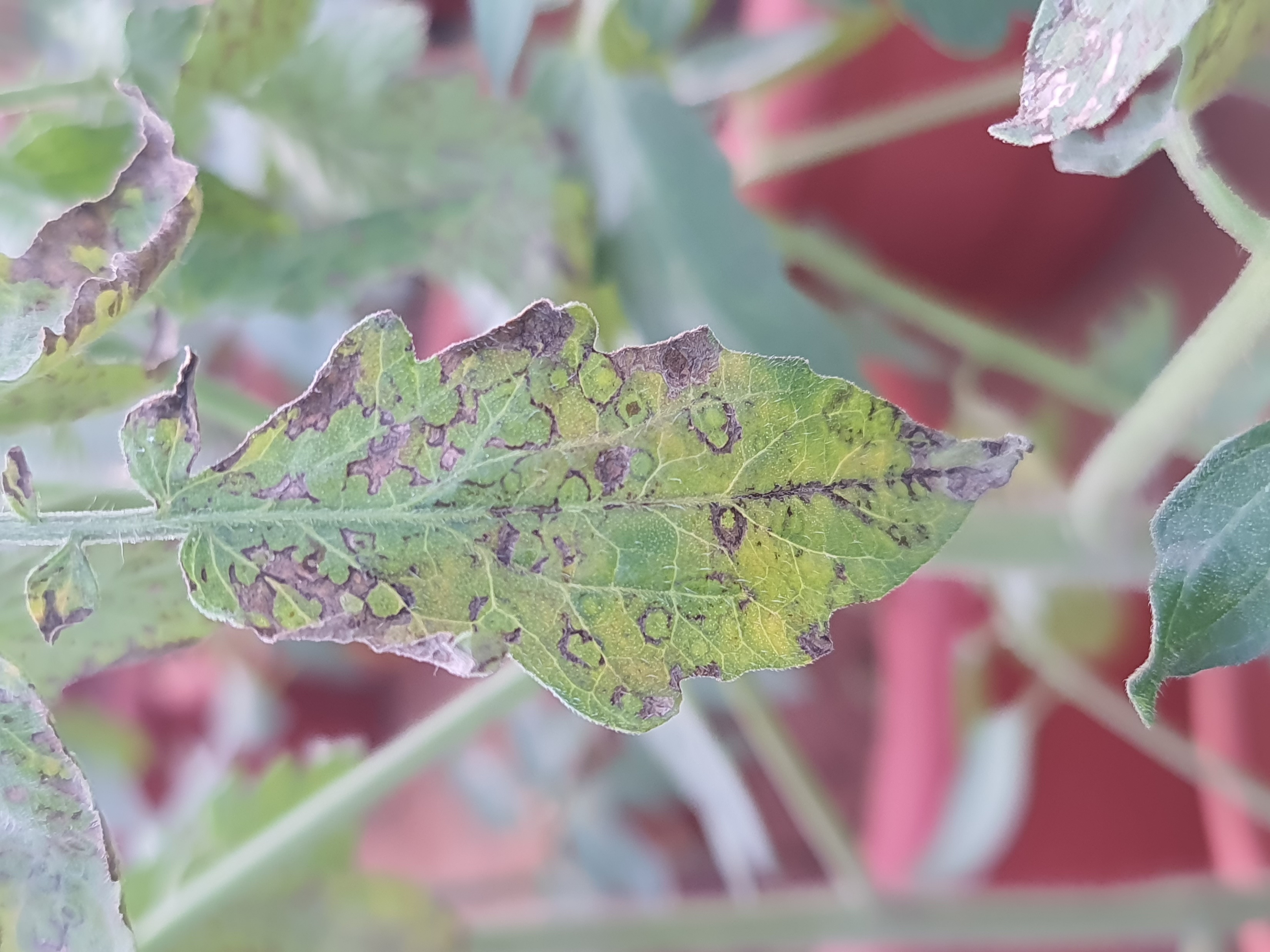


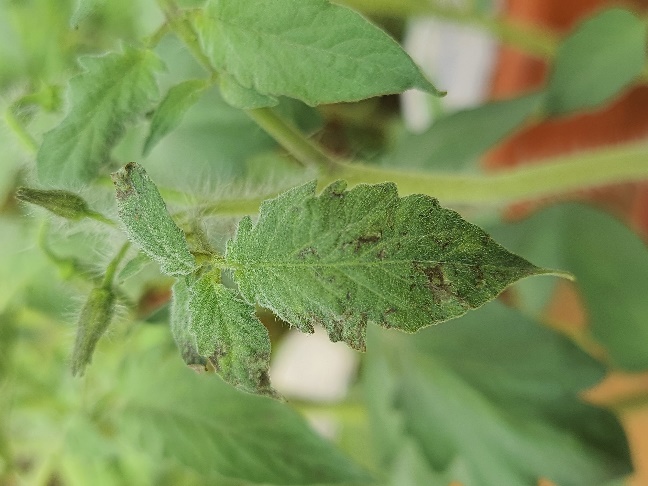


**0**

**1**

**4**

**3**

**2**

0. No symptom,1. Initiation of necrosis, 2. Moderate necrosis, 3. Severe and spread of necrosis

4. Complete drying of leaves and Stem necrosis

**Table S1. GPS data of GBNV infected sample collected from various district (Coimbatore,**

**and Krishnagiri)**

| **District** | **Location Name** | **GIS position** | **Variety** |
| --- | --- | --- | --- |
| Coimbatore | Devarayapuram | 10.999359^o^E, 76.794404^o^N | Darsh gold and Surekha |
|  | Booluvampatti | 10.994573^o^E, 76.797305^o^N | Surekha |
|  | Tholampalayam | 11.236181^o^E, 76.936648^o^N | Sago |
|  | Marchinaickenpalayam) | 10.6010.607396^o^E, 76.91846^o^N.9184^0^ E |  |
| Krishnagiri | Hosur | 12.641325^o^E,77.831741^o^N |  |
|  |  |  | Shivam |

**Table. S2. Assessing the copy number of GBNV Nucleocapsid gene (N) in bioagents treated**

**cowpea through real time PCR**

| **Bacterial endophytes** | **0 h** | **24 h** | **48 h** | **72 h** | **96 h** |
| --- | --- | --- | --- | --- | --- |
| *Myroides odorotimimus* (YEB RT3) | 7.4×10^5^ | 5.3×10^5^ | 5.7×10^5^ | 5.2×10^6^ | 4.5×10^7^ |
| ***Bacillus tequilensis* (NBL 6)** | **1.1×10^5^** | **4.5×10^5^** | **4.7×10^5^** | **3.4×10^6^** | **3.6×10^6^** |
| ***Bacilus velezensis*(VB7)** | **1.0×10^5^** | **4.0×10^5^** | **4.8×10^5^** | **2.8×10^6^** | **3.5×10^6^** |
| ***Bacillus licheniformis* (Soya 1)** | **1.3×10^5^** | **3.7×10^5^** | **4.5×10^5^** | **3.5×10^6^** | **2.4×10^7^** |
| *Bacillus sonorenis* (KMR3) | 2.8×10^5^ | 5.1×10^5^ | 5.3×10^5^ | 3.7×10^6^ | 4.3×10^7^ |
| Inoculated control | 7.7×10^5^ | 5.8×10^5^ | 3.8×10^6^ | 1.8×10^7^ | 1.2×10^8^ |
